# Clinical Characteristics, Microbial Profile, and Factors Associated with Recurrent Urinary Tract Infections in Patients with Diabetes Mellitus: A Retrospective Cohort Study

**DOI:** 10.7759/cureus.115733

**Published:** 2026-09-03

**Authors:** Mya Ya Minn, Asma Arshad, Humna Khan, Saima Shabir, Rijja Feroz, Najeeb Ullah, Muhammad Zain Ul Abideen, Daud Gul

**Affiliations:** 1 Nephrology, Basildon Hospital, Basildon, GBR; 2 Obstetrics and Gynaecology, Fatima Memorial Hospital, Lahore, PAK; 3 Internal Medicine, Fatima Memorial Hospital, Lahore, PAK; 4 Neurology, Sheikh Zayed Hospital, Rahim Yar Khan, PAK; 5 Internal Medicine, Fatima Jinnah Medical University, Lahore, PAK; 6 Internal Medicine, Tehsil Headquarter Hospital, Fateh Jang, PAK; 7 Internal Medicine, Bridgeport Medical Clinic, Waterloo, CAN

**Keywords:** diabetes mellitus, diabetic nephropathy, microbial profile, recurrent urinary tract infections, urinary catheterization

## Abstract

Background and objective

Recurrent urinary tract infection (UTI) is a common and clinically important complication among patients with type 2 diabetes mellitus (T2DM), contributing to increased morbidity, antimicrobial resistance, and healthcare utilization. This study aimed to evaluate the clinical characteristics, microbial profile, and factors associated with recurrent UTIs among patients with T2DM.

Methodology

This retrospective comparative observational study was conducted at the Fatima Memorial Hospital, Lahore, Pakistan, using medical records of patients managed between March 2023 and March 2026. A total of 455 adult patients with T2DM and at least one culture-confirmed UTI were included. Patients were categorized into recurrent UTI (n = 196) and non-recurrent UTI (n = 259) groups. Demographic characteristics, clinical features, microbiological findings, laboratory parameters, and potential associated factors were extracted from medical records. Univariable and multivariable binary logistic regression analyses were performed to identify factors independently associated with recurrent UTI.

Results

Of the 455 patients, 196 (43.1%) had recurrent UTI and 259 (56.9%) had non-recurrent UTI. Patients with recurrent UTI were older, had a longer duration of diabetes, had higher HbA1c levels, and a greater proportion were female. Diabetic nephropathy, nephrolithiasis, previous antibiotic use, and urinary catheterization were more common in the recurrent UTI group. *Escherichia coli* was the predominant uropathogen in both groups, while multidrug-resistant organisms (MDROs) were significantly more frequent among patients with recurrent UTI (98 (50.0%) vs. 75 (29.0%); p <0.001). In multivariable analysis, poor glycemic control (adjusted OR 2.41, 95% CI 1.58-3.67), previous antibiotic use (adjusted OR 2.08, 95% CI 1.36-3.19), diabetic nephropathy (adjusted OR 1.76, 95% CI 1.12-2.78), urinary catheterization (adjusted OR 2.69, 95% CI 1.57-4.61), and MDROs (adjusted OR 1.82, 95% CI 1.20-2.76) were independently associated with recurrent UTI.

Conclusion

Recurrent UTI among patients with T2DM was associated with poor glycemic control, diabetic nephropathy, previous antibiotic exposure, urinary catheterization, and infection with MDROs. These findings may facilitate early identification of high-risk patients and support targeted strategies for optimizing glycemic control, antimicrobial stewardship, and prevention of recurrent UTI.

## Introduction

Urinary tract infections (UTIs) are among the most common bacterial infections encountered in clinical practice and represent a major cause of morbidity, healthcare utilization, and antibiotic consumption worldwide [1]. Approximately 150 million people develop UTIs annually, resulting in substantial healthcare costs and frequent hospitalizations. Although most UTIs respond well to antimicrobial therapy, recurrence is common, particularly among individuals with underlying conditions such as type 2 diabetes mellitus (T2DM) [2]. Recurrent UTI is commonly defined as two or more culture-confirmed episodes within six months or three or more episodes within one year and is associated with repeated antibiotic exposure, antimicrobial resistance, reduced quality of life, and an increased risk of complications [3].

Diabetes mellitus is a major global public health challenge, affecting an estimated 589 million adults aged 20 to 79 years worldwide, with projections reaching 853 million by 2050. Pakistan has one of the highest diabetes burdens globally, with approximately 34.5 million affected adults and an estimated prevalence of 31.4%, according to the International Diabetes Federation Diabetes Atlas 2025 [4]. Patients with diabetes have been reported to have a 1.5- to three-fold higher risk of developing UTIs than the general population [5]. Local studies report UTI prevalence ranging from 20% to 40% among individuals with diabetes, with recurrent infections reported more frequently than in non-diabetic populations. Factors that may contribute to increased susceptibility to UTI in diabetes include persistent hyperglycemia and glycosuria, impaired neutrophil function, altered cellular immunity, autonomic neuropathy with incomplete bladder emptying, microvascular disease, and structural abnormalities of the urinary tract [6,7].

Uropathogenic *Escherichia coli *remains the predominant causative organism in both uncomplicated and recurrent UTIs, although other pathogens such as *Klebsiella pneumoniae*, *Proteus mirabilis*, *Pseudomonas aeruginosa*, *Enterococcus faecalis*, and *Staphylococcus saprophyticu*s are also frequently isolated [8]. Furthermore, multidrug-resistant organisms (MDROs), including extended-spectrum β-lactamase (ESBL)-producing Gram-negative bacteria, are increasingly reported among patients with diabetes, potentially limiting empirical antimicrobial options and complicating treatment [9].

Several demographic and clinical factors have been associated with recurrent UTIs in patients with diabetes, including female sex, older age, longer duration of diabetes, poor glycemic control, diabetic nephropathy, obesity, urinary catheterization, urinary tract obstruction, previous UTI, and nephrolithiasis [10]. In addition, the microbial spectrum and antimicrobial resistance patterns of UTIs may vary according to geographical region, healthcare setting, and local antibiotic prescribing practices, highlighting the importance of continuous surveillance to inform empirical treatment and antimicrobial stewardship [11,12]. However, data describing the clinical characteristics, microbial profile, and factors associated with recurrent UTIs among patients with diabetes remain limited in Pakistan. Therefore, this study was conducted to compare the clinical characteristics and microbial profile of diabetic patients with recurrent and non-recurrent UTIs among patients with T2DM and to identify independent factors associated with recurrent UTI (defined as ≥2 symptomatic culture-confirmed episodes within six months or ≥3 episodes within 12 months using multivariable logistic regression) after adjustment for clinically relevant confounders.

## Materials and methods

Study design and setting

This retrospective comparative observational study was conducted at Fatima Memorial Hospital, Lahore, Pakistan, using electronic medical and microbiology records of patients managed between March 2023 and March 2026. Fatima Memorial Hospital is a tertiary-care teaching hospital providing outpatient, emergency, inpatient, and specialist medical services. Patients were identified through the hospital electronic medical record system and microbiology laboratory database. Eligible patients managed through the medical outpatient department, emergency department, and inpatient medical wards were considered for inclusion. Patients evaluated by specialized urology services were also eligible when relevant clinical and microbiological records were available within the hospital electronic medical record system.

A total of 1,184 potentially relevant records were identified during the study period. After removal of duplicate records and application of the predefined inclusion and exclusion criteria, 455 adult patients with T2DM and at least one symptomatic, culture-confirmed UTI were included in the final analysis. All eligible patients meeting the predefined selection criteria during the study period were included. The inclusion and exclusion criteria are featured in Table 1.

**Table 1:** Inclusion and exclusion criteria for participants UTI: Urinary tract infection, T2DM: Type 2 diabetes mellitus

Inclusion criteria	Exclusion criteria
Age ≥18 years	Type 1 diabetes mellitus
Diagnosed T2DM	Pregnancy
At least one symptomatic culture-confirmed UTI during the study period	Congenital urinary tract abnormalities
Complete demographic, clinical, laboratory, and microbiological records	Active malignancy
For recurrent UTI group: ≥2 symptomatic culture-confirmed UTIs within six months or ≥3 within 12 months	Immunodeficiency disorders
For non-recurrent UTI group: an index symptomatic culture-confirmed UTI without fulfilling recurrent UTI criteria during the available observation period	Current immunosuppressive therapy
_____	Incomplete clinical or microbiological records

The study was designed to evaluate factors associated with recurrent UTI among patients who had already experienced a culture-confirmed UTI and was not intended to estimate the prevalence of UTI among the general diabetic population. Because this study involved a retrospective review of existing anonymized medical and microbiological records without direct participant intervention, the requirement for informed consent was waived by the Institutional Review Board/Ethics Committee of Fatima Memorial Hospital.

Study population

The first eligible symptomatic culture-confirmed UTI documented during the study period was considered the index UTI. Patients were subsequently classified according to the occurrence of further documented symptomatic culture-confirmed UTI episodes. Group A comprised those with recurrent UTI, i.e., patients who developed at least two symptomatic culture-confirmed UTI episodes within six months or at least three symptomatic culture-confirmed UTI episodes within 12 months. Group B included patients with non-recurrent UTI, namely those with an index symptomatic culture-confirmed UTI who did not develop a subsequent documented symptomatic culture-confirmed UTI, thereby fulfilling the predefined criteria for recurrence during their available observation period.

The dates of individual UTI episodes were determined from documented clinical symptoms and/or urine specimen collection dates recorded in the electronic medical and microbiology records. The interval between episodes was calculated from the date of the preceding episode to the date of the subsequent episode. When the exact date of symptom onset was unavailable, the urine specimen collection date was used as the reference date.

A total of 1,184 potentially relevant records were identified through the electronic medical record system and microbiology laboratory database during the study period. Duplicate records were removed, and the remaining records were screened according to the predefined eligibility criteria. Records were excluded because of absence of a symptomatic culture-confirmed UTI (n = 386), asymptomatic bacteriuria or culture contamination (n = 97), type 1 diabetes mellitus (n = 42), age <18 years (n = 31), pregnancy (n = 18), congenital urinary tract abnormalities (n = 11), active malignancy (n = 16), immunodeficiency disorders (n = 8), current immunosuppressive therapy (n = 13), or incomplete clinical or microbiological records (n = 71). After application of the eligibility criteria, 455 patients were included in the final analysis. Of these, 196 patients fulfilled the predefined criteria for recurrent UTI, while 259 were classified as having non-recurrent UTI. The patient selection process is presented in the Strengthening the Reporting of Observational Studies in Epidemiology (STROBE) flow diagram (Figure 1).

**Figure 1:**
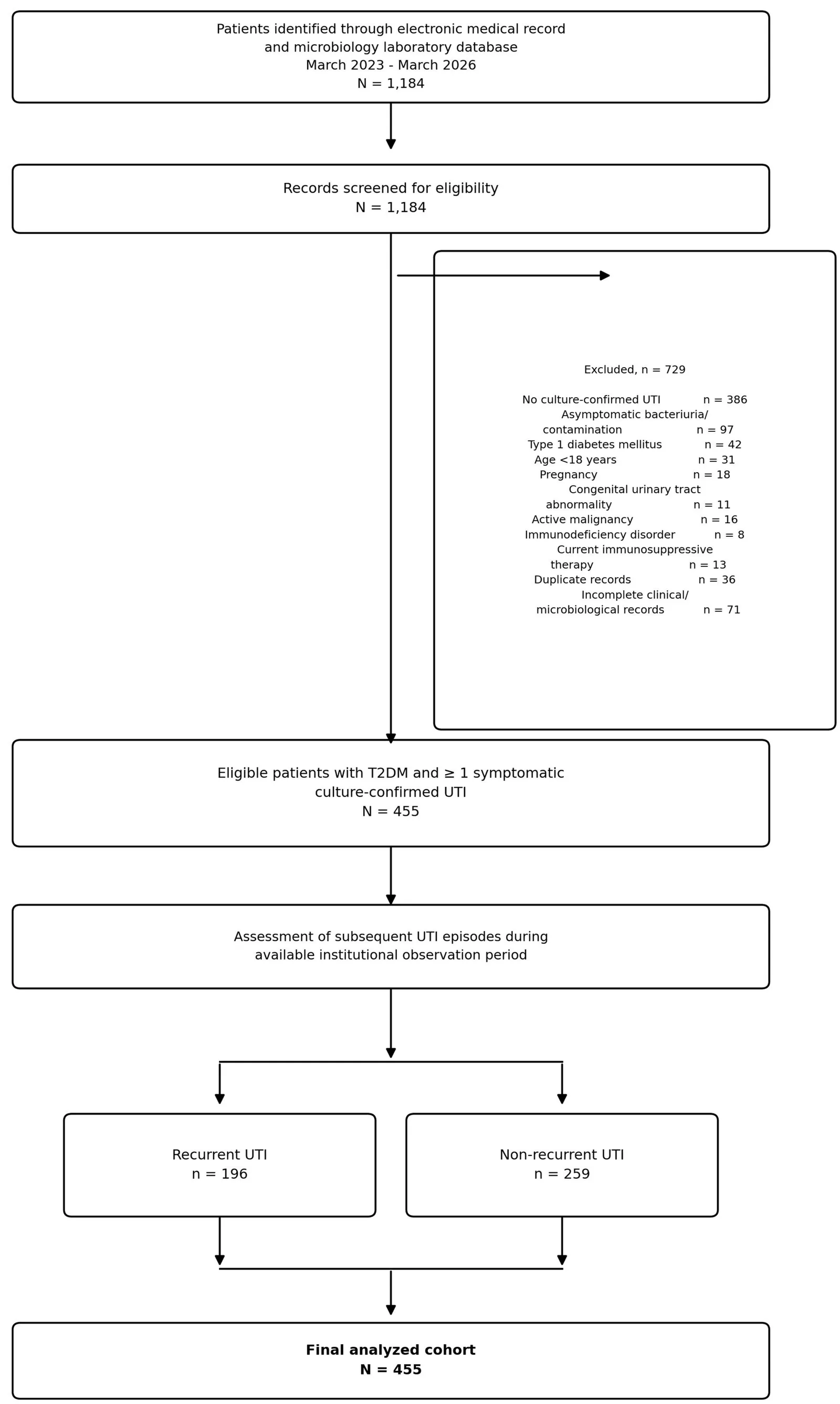
STROBE flow diagram of patient selection STROBE: Strengthening the Reporting of Observational Studies in Epidemiology, UTI: Urinary tract infection

Definition of symptomatic UTI and positive urine culture

A symptomatic UTI was defined as the presence of at least one urinary or systemic clinical feature compatible with UTI, including dysuria, increased urinary frequency, urinary urgency, suprapubic pain, flank pain, or fever, together with a clinically significant positive urine culture. Urine specimens were collected before initiation of antimicrobial therapy whenever clinically feasible according to hospital protocol. A positive urine culture was defined as isolation of a single clinically relevant urinary pathogen at a concentration of ≥10⁵ colony-forming units (CFU)/mL in a properly collected midstream urine specimen, consistent with the laboratory criteria for significant bacteriuria used during the study period.

Only cultures considered clinically significant by the hospital microbiology laboratory were included. Cultures reported as mixed bacterial growth or contamination were not considered positive cultures for classification of UTI. Asymptomatic bacteriuria was excluded; therefore, a positive urine culture in the absence of compatible urinary or systemic symptoms was not counted as a UTI episode.

Definition and ascertainment of recurrent UTI

Recurrent UTI was defined as at least two symptomatic culture-confirmed UTI episodes within six months or at least three symptomatic culture-confirmed UTI episodes within 12 months. To distinguish recurrent infection from persistent infection or treatment failure, a subsequent positive urine culture was considered a separate UTI episode only when there was documented clinical resolution or substantial improvement of the preceding infection. Repeat positive cultures obtained during the same symptomatic episode or during ongoing antimicrobial treatment without documented clinical resolution were considered part of the same infection episode and were not counted as separate recurrences. The interval between separate UTI episodes was determined from the dates of documented clinical symptoms and/or urine specimen collection recorded in the electronic medical and microbiology records. When the exact date of symptom onset was unavailable, the urine specimen collection date was used.

Follow-up and ascertainment of non-recurrent UTI

Available outpatient, emergency department, inpatient, and microbiology records were reviewed from the index UTI through the end of the study observation period in March 2026 to identify subsequent symptomatic culture-confirmed UTI episodes. Patients were classified as having a non-recurrent UTI when no subsequent documented symptomatic culture-confirmed UTI fulfilling the predefined criteria for recurrence was identified during their available institutional observation period. Patients with insufficient clinical or microbiological documentation to determine recurrence status were excluded.

For patients whose index UTI occurred sufficiently early in the study period, recurrence was assessed for up to 12 months after the index infection. For patients whose index UTI occurred during the final 12 months of the study period, recurrence was assessed only during the available observation period up to March 2026. The median available follow-up duration was 14 months. Because this was a retrospective single-center study, UTI episodes diagnosed or treated outside Fatima Memorial Hospital could not be reliably captured. Therefore, absence of a documented recurrence indicates absence of a recorded recurrence within the available institutional observation period and does not necessarily indicate that the patient experienced no UTI recurrence outside the hospital.

Data collection

Medical records were reviewed using a standardized data collection proforma. Demographic variables included age, sex, BMI, duration of diabetes, smoking status, and relevant comorbidities. Clinical variables included dysuria, increased urinary frequency, urgency, suprapubic pain, fever, flank pain, previous antibiotic exposure, previous hospitalization, urinary catheterization, nephrolithiasis, urinary tract obstruction, diabetic nephropathy, and glycemic control. Previous antibiotic exposure was defined as documented systemic antibacterial therapy received within 90 days before the index UTI. Antibiotics prescribed specifically for treatment of the index UTI were not considered previous antibiotic exposure. For patients with documented prior antibiotic therapy, the antibiotic class, indication, duration of therapy, and interval between completion of antibiotic therapy and the index UTI were recorded when available. Antibiotic indications were categorized as UTI, respiratory tract infection, skin/soft-tissue infection, gastrointestinal infection, or other documented bacterial infection. The interval between the most recent antibiotic exposure and the index UTI was calculated in days. Antibiotic exposure occurring after the index UTI was not included in the baseline exposure variable.

Among patients with documented previous antibiotic exposure, the most frequently recorded antibiotic classes were fluoroquinolones, third-generation cephalosporins, penicillin/beta-lactam agents, trimethoprim-sulfamethoxazole, nitrofurantoin, and carbapenems. Most antibiotic courses were prescribed for suspected or documented bacterial infections and were completed within 14 days. These variables were evaluated descriptively to characterize prior antimicrobial exposure but were not entered separately into the primary multivariable regression model because of the limited number of exposed patients and potential collinearity among individual antibiotic classes.

The HbA1c was recorded using the most recent measurement obtained within three months before or at the time of the index UTI. Measurements obtained after the index UTI were not used to define baseline glycemic control. Poor glycemic control was defined as HbA1c ≥8%.

Microbiological assessment

Microbiological data were obtained from hospital microbiology laboratory records. Urine specimens were collected using standard hospital procedures before initiation of antibiotic therapy whenever feasible. Urine routine examination, culture, organism identification, and antimicrobial susceptibility testing were performed according to standard laboratory protocols. A urine culture was considered significant when a single clinically relevant urinary pathogen was isolated at ≥10⁵ CFU/mL in a properly collected midstream urine specimen. Cultures reported as mixed bacterial growth or contamination were excluded from the analysis.

Antimicrobial susceptibility testing was performed according to the Clinical and Laboratory Standards Institute (CLSI) Performance Standards for Antimicrobial Susceptibility Testing (M100), 35th edition (2025) [13]. The microbiology laboratory followed established internal quality-control procedures, including the use of appropriate quality-control/reference strains and standardized procedures for bacterial culture, organism identification, and antimicrobial susceptibility testing. For the primary microbiological analysis, only the isolate obtained from the index UTI was used, thereby avoiding statistical duplication of patients and preventing microbiological characteristics arising during subsequent recurrent episodes from being treated as baseline predictors.

Multidrug-resistant organisms were defined according to the internationally accepted criteria proposed by Magiorakos et al., in which multidrug resistance refers to acquired non-susceptibility to at least one antimicrobial agent in three or more antimicrobial categories [14]. The MDRO status was determined from the antimicrobial susceptibility profile of the index UTI isolate. Resistance phenotypes, including ESBL-producing *Enterobacterales*, carbapenem-resistant *Enterobacterales* (CRE), multidrug-resistant *P. aeruginosa*, and multidrug-resistant *Acinetobacter* species, were classified as MDROs when they fulfilled the predefined multidrug-resistance criterion. The MDRO status was therefore established before ascertainment of subsequent UTI recurrence and was not derived from cultures obtained during recurrent episodes.

Risk factors and clinical variables

Potential factors associated with recurrent UTI included age, sex, BMI, duration of diabetes, smoking status, hypertension, diabetic nephropathy, nephrolithiasis, urinary tract obstruction, previous hospitalization, previous antibiotic exposure, urinary catheterization, poor glycemic control, and MDRO status based on the index UTI isolate. Several established risk factors for recurrent UTI, including post-void residual urine volume, menopausal status, benign prostatic hyperplasia, sodium-glucose cotransporter-2 (SGLT2) inhibitor use, urinary incontinence, neurogenic bladder, and detailed structural urinary tract abnormalities, were not consistently documented in the retrospective medical records and therefore were not included in the regression analysis. The absence of these variables represents an important limitation and may have resulted in residual confounding.

Outcome definition

The primary outcome was recurrent UTI, defined as at least two symptomatic culture-confirmed UTI episodes within six months or at least three symptomatic culture-confirmed UTI episodes within 12 months, with documented clinical resolution or substantial improvement of the preceding infection before a subsequent episode was considered a recurrence. The primary analysis compared patients with recurrent UTI with patients who had an index symptomatic culture-confirmed UTI but did not develop a subsequent documented UTI fulfilling the predefined criteria for recurrence during their available institutional observation period.

Statistical analysis

Data were entered and analyzed using SPSS Statistics version 26.0 (IBM Corp., Armonk, NY, USA). Continuous variables were assessed for distributional normality using the Shapiro-Wilk test. Variables demonstrating an approximately normal distribution were summarized as mean ± SD, whereas non-normally distributed variables were summarized as median with IQR. Categorical variables were summarized as frequencies and percentages.

Continuous variables were compared between recurrent and non-recurrent UTI groups using the independent-samples t-test for normally distributed variables or the Mann-Whitney U test for non-normally distributed variables. Categorical variables were compared using Pearson's chi-square test or Fisher's exact test, as appropriate.

Binary logistic regression was performed with recurrent UTI as the dependent variable. Candidate predictors included age, sex, diabetes duration, poor glycemic control (HbA1c ≥8%), diabetic nephropathy, previous antibiotic exposure, urinary catheterization, nephrolithiasis, and MDRO status based on the index UTI isolate. Candidate variables were selected a priori based on clinical relevance and previously reported associations with recurrent UTI. Variables demonstrating an association at p<0.05 in univariable analysis were considered for multivariable analysis together with clinically relevant potential confounders.

Multicollinearity among candidate predictors was assessed before construction of the final multivariable model using variance inflation factors (VIFs) and tolerance values. A VIF <5 and tolerance >0.20 were considered indicative of acceptable levels of multicollinearity. No clinically important multicollinearity was identified among the variables included in the final model. Missing data were handled using complete-case analysis. Patients with incomplete demographic, clinical, laboratory, or microbiological records were excluded during the predefined eligibility assessment.

Model fit was assessed using the Hosmer-Lemeshow goodness-of-fit test. Adjusted odds ratios (aORs) with corresponding 95% CIs were reported. Statistical tests were two-sided, and a p-value <0.05 was considered statistically significant.

## Results

A total of 455 patients with T2DM and at least one symptomatic culture-confirmed UTI were included in the final analysis. Of these, 196 (43.1%) had recurrent UTI and 259 (56.9%) had non-recurrent UTI. Patients with recurrent UTI were older than those with non-recurrent UTI (59.1 ± 10.8 vs. 55.1 ± 11.6 years; p <0.001). Female sex was more common among patients with recurrent UTI than among those with non-recurrent UTI (72.4% vs. 60.2%; p=0.008). Patients with recurrent UTI also had a longer duration of diabetes (11.2 ± 5.4 vs. 8.4 ± 4.7 years; p<0.001) and higher mean HbA1c levels (9.0 ± 1.5% vs. 7.9 ± 1.5%; p<0.001). Mean BMI was slightly higher in the recurrent UTI group, although the difference was not statistically significant (28.7 ± 4.6 vs. 27.9 ± 4.4 kg/m²; p=0.062). Diabetic nephropathy (34.2% vs. 19.7%; p<0.001), nephrolithiasis (18.9% vs. 10.4%; p=0.010), previous antibiotic exposure (74.0% vs. 50.6%; p<0.001), and urinary catheterization (26.5% vs. 11.6%; p<0.001) were significantly more frequent among patients with recurrent UTI. The prevalence of hypertension did not differ significantly between groups (59.7% vs. 52.1%; p=0.108) (Table 2).

**Table 2:** Comparison of baseline demographic and clinical characteristics between recurrent and non-recurrent UTI groups (total n = 455) Data are presented as mean ± SD for continuous variables and number (percentage) for categorical variables. Continuous variables demonstrated approximately normal distributions based on Shapiro-Wilk testing; therefore, independent-samples t-tests were used for continuous variables. Categorical variables were compared using Pearson's chi-square test or Fisher's exact test, as appropriate. UTI: Urinary tract infection

Variable	Recurrent UTI (n=196)	Non-recurrent UTI (n=259)	p-value
Age (years), mean ± SD	59.1 ± 10.8	55.1 ± 11.6	<0.001
Male	54 (27.6%)	103 (39.8%)	0.008
Female	142 (72.4%)	156 (60.2%)	0.008
Duration of diabetes (years), mean ± SD	11.2 ± 5.4	8.4 ± 4.7	<0.001
HbA1c (%), mean ± SD	9.0 ± 1.5	7.9 ± 1.5	<0.001
BMI (kg/m²), mean ± SD	28.7 ± 4.6	27.9 ± 4.4	0.062
Hypertension	117 (59.7%)	135 (52.1%)	0.108
Diabetic nephropathy	67 (34.2%)	51 (19.7%)	<0.001
Nephrolithiasis	37 (18.9%)	27 (10.4%)	0.010
Previous antibiotic use	145 (74.0%)	131 (50.6%)	<0.001
Urinary catheterization	52 (26.5%)	30 (11.6%)	<0.001

Among the 455 patients, 276 (60.7%) had documented systemic antibiotic exposure within 90 days before the index UTI. Fluoroquinolones were the most frequently used antibiotic class (92/276, 33.3%), followed by third-generation cephalosporins (71/276, 25.7%) and penicillin/beta-lactam agents (58/276, 21.0%). The most common indication for antibiotic therapy was a previous UTI (168/276, 60.9%), followed by respiratory tract infections (48/276, 17.4%) and skin and soft-tissue infections (25/276, 9.1%). Most antibiotic courses lasted four to seven days (151/276, 54.7%), followed by eight to 14 days (61/276, 22.1%). Regarding the interval between completion of antibiotic therapy and the index UTI, 113 patients (40.9%) had received antibiotics within 30 days, 91 (33.0%) within 31 to 60 days, and 72 (26.1%) within 61 to 90 days (Table 3).

**Table 3:** Characteristics of previous antibiotic exposure

Variable		Total exposed (n=276)
Antibiotic class	Fluoroquinolones	92 (33.3%)
	Third-generation cephalosporins	71 (25.7%)
	Penicillin/beta-lactam agents	58 (21.0%)
	Trimethoprim-sulfamethoxazole	29 (10.5%)
	Nitrofurantoin	18 (6.5%)
	Carbapenems	8 (2.9%)
Indication for antibiotic therapy	Urinary tract infection	168 (60.9%)
	Respiratory tract infection	48 (17.4%)
	Skin/soft-tissue infection	25 (9.1%)
	Gastrointestinal infection	12 (4.3%)
	Other documented bacterial infection	23 (8.3%)
Duration of therapy	≤3 days	44 (15.9%)
	4-7 days	151 (54.7%)
	8-14 days	61 (22.1%)
	>14 days	20 (7.2%)
Interval from antibiotic completion to index UTI	≤30 days	113 (40.9%)
	31-60 days	91 (33.0%)
	61-90 days	72 (26.1%)

Dysuria was the most common presenting symptom in both groups, occurring in 171 (87.2%) of patients with recurrent UTI and 211 (81.5%) of those with non-recurrent UTI; however, this difference was not statistically significant (p = 0.098). Similarly, increased urinary frequency was more common among patients with recurrent UTI, although the difference did not reach statistical significance: 157 (80.1%) vs. 189 (73.0%); p = 0.077. In contrast, urgency, suprapubic pain, fever, and flank pain were significantly more frequent in the recurrent UTI group (Table 4).

**Table 4:** Comparison of clinical presentation between recurrent and non-recurrent UTI groups Data are presented as numbers (percentages). Comparisons between groups were performed using Pearson's chi-square test. All chi-square tests were performed with 1 degree of freedom (df = 1). Statistical significance was defined as a two-tailed p-value <0.05. UTI: Urinary tract infection

Clinical feature	Recurrent UTI (n = 196)	Non-recurrent UTI (n = 259)	χ²	p-value
Dysuria	171 (87.2%)	211 (81.5%)	2.73	0.098
Increased urinary frequency	157 (80.1%)	189 (73.0%)	3.12	0.077
Urgency	137 (69.9%)	154 (59.5%)	5.22	0.022
Suprapubic pain	91 (46.4%)	93 (35.9%)	5.11	0.024
Fever	72 (36.7%)	67 (25.9%)	6.18	0.013
Flank pain	52 (26.5%)	44 (17.0%)	6.07	0.014

*Escherichia coli *was the predominant uropathogen in both recurrent and non-recurrent UTI groups (48.0% vs. 52.9%; p = 0.301). No statistically significant differences were observed in the distribution of other isolated organisms between the groups. However, MDROs were isolated significantly more frequently in patients with recurrent UTI than in those with non-recurrent UTI (50.0% vs. 29.0%; p < 0.001) (Table 5).

**Table 5:** Comparison of microbial profile between recurrent and non-recurrent UTI groups Data are presented as numbers (percentages). Comparisons between groups were performed using Pearson's chi-square test. All chi-square tests were performed with 1 degree of freedom (df = 1). Statistical significance was defined as a two-tailed p-value <0.05. MDRO: Multidrug-resistant organism, UTI: Urinary tract infection

Isolated organism	Recurrent UTI (n = 196)	Non-recurrent UTI (n = 259)	χ²	p-value
E. coli	94 (48.0%)	137 (52.9%)	1.07	0.301
K. pneumoniae	44 (22.4%)	47 (18.1%)	1.29	0.256
E. faecalis	22 (11.2%)	20 (7.7%)	1.63	0.201
P. aeruginosa	20 (10.2%)	15 (5.8%)	3.04	0.081
P. mirabilis	8 (4.1%)	21 (8.1%)	3.00	0.083
*Candida* species	7 (3.6%)	11 (4.2%)	0.13	0.722
S. saprophyticus	1 (0.5%)	8 (3.1%)	3.78	0.052
MDRO	98 (50.0%)	75 (29.0%)	21.11	<0.001

Univariable logistic regression analysis demonstrated that age ≥60 years, female sex, diabetes duration ≥10 years, poor glycemic control (HbA1c ≥8%), diabetic nephropathy, previous antibiotic use, urinary catheterization, nephrolithiasis, and infection with MDROs were significantly associated with recurrent UTI (Table 5). Poor glycemic control showed the strongest association in the univariable analysis (crude OR 3.18; 95% CI: 2.10-4.82; p <0.001), followed by previous antibiotic use (crude OR 2.78; 95% CI: 1.86-4.15; p <0.001) and urinary catheterization (crude OR 2.76; 95% CI: 1.69-4.50; p <0.001) (Table 6).

**Table 6:** Univariable logistic regression analysis of factors associated with recurrent UTI Crude ORs were estimated using univariable binary logistic regression. Variables with p<0.05 in univariable analysis and clinically relevant variables were considered for inclusion in the multivariable model. Statistical significance was defined as a two-tailed p-value <0.05. MDRO: Multidrug-resistant organism, UTI: Urinary tract infection

Risk factor	Crude OR	95% CI	p-value
Age ≥60 years	1.67	1.15–2.43	0.007
Female gender	1.74	1.17–2.58	0.006
Diabetes duration ≥10 years	2.24	1.53–3.29	<0.001
Poor glycemic control, HbA1c ≥8%	3.18	2.10–4.82	<0.001
Diabetic nephropathy	2.12	1.41–3.20	<0.001
Previous antibiotic use	2.78	1.86–4.15	<0.001
Urinary catheterization	2.76	1.69–4.50	<0.001
Nephrolithiasis	2.00	1.18–3.39	0.010
MDRO	2.45	1.67–3.60	<0.001

In the multivariable logistic regression model, eight prespecified candidate predictors were entered simultaneously: age ≥60 years, female sex, diabetes duration ≥10 years, poor glycemic control (HbA1c ≥8%), diabetic nephropathy, previous antibiotic exposure, urinary catheterization, and nephrolithiasis, together with MDRO status from the index UTI isolate. No clinically important multicollinearity was identified among the included predictors, with all VIF values remaining <5 and tolerance values >0.20.

Poor glycemic control remained independently associated with recurrent UTI (aOR 2.41, 95% CI 1.58-3.67; p<0.001). Previous antibiotic exposure was also independently associated with recurrence (aOR 2.08, 95% CI 1.36-3.19; p=0.001), as were diabetic nephropathy (aOR 1.76, 95% CI 1.12-2.78; p=0.014), urinary catheterization (aOR 2.69, 95% CI 1.57-4.61; p<0.001), and MDRO status (aOR 1.82, 95% CI 1.20-2.76; p=0.005). Female sex, diabetes duration ≥10 years, and nephrolithiasis were not independently associated with recurrent UTI after adjustment (Table 7). The final model demonstrated acceptable calibration (Hosmer-Lemeshow goodness-of-fit p=0.42), with a Nagelkerke R² of 0.28, indicating moderate discriminatory performance.

**Table 7:** Multivariable binary logistic regression analysis of independent predictors of recurrent UTI The aORs with 95% CIs were estimated using multivariable binary logistic regression after adjustment for potential confounding variables. Statistical significance was defined as a two-tailed p-value <0.05. aOR: Adjusted odds ratio, MDRO: Multidrug-resistant organism, UTI: Urinary tract infection

Predictor	Adjusted OR	95% CI	p-value
Poor glycemic control, HbA1c ≥8%	2.41	1.58–3.67	<0.001
Previous antibiotic use	2.08	1.36–3.19	0.001
Diabetic nephropathy	1.76	1.12–2.78	0.014
Urinary catheterization	2.69	1.57–4.61	<0.001
MDRO	1.82	1.20–2.76	0.005
Female gender	1.43	0.93–2.20	0.103
Diabetes duration ≥10 years	1.49	0.98–2.28	0.064
Nephrolithiasis	1.38	0.78–2.45	0.269

## Discussion

This retrospective comparative study evaluated the clinical characteristics, microbial profile, and factors associated with recurrent UTI among patients with T2DM. Patients with recurrent UTI were older, more frequently female, and had a longer duration of diabetes, a poorer glycemic control, and a higher prevalence of diabetic nephropathy, previous antibiotic exposure, urinary catheterization, and nephrolithiasis than those with non-recurrent UTI. After adjustment for potential confounding variables, poor glycemic control, previous antibiotic exposure, diabetic nephropathy, urinary catheterization, and infection with MDROs remained independently associated with recurrent UTI. Given the retrospective observational design, these findings indicate associations and should not be interpreted as evidence of causal relationships.

Female patients constituted a greater proportion of the recurrent UTI group than the non-recurrent group. Although female sex was significantly associated with recurrent UTI in the univariable analysis, this association was no longer statistically significant after multivariable adjustment. This attenuation suggests that the observed unadjusted association may partly reflect differences in other clinical characteristics, including glycemic control, diabetic nephropathy, previous antibiotic exposure, and urinary catheterization. The higher frequency of UTI among women has been reported in previous studies and may be related to anatomical and physiological characteristics, including a shorter urethra, proximity of the urethral meatus to the perineum, hormonal influences, and differences in bacterial colonization [15]. These mechanisms may contribute to susceptibility to UTI, although their specific contribution to recurrence could not be established in the present study.

Patients with recurrent UTIs also had a longer duration of diabetes than those with non-recurrent infections. Although diabetes duration was significantly associated with recurrence in the univariable analysis, the association was attenuated after adjustment for other variables and did not remain statistically significant in the final model. Longer diabetes duration may be accompanied by complications such as autonomic neuropathy, impaired bladder emptying, microvascular disease, and altered immune function, which have been associated with susceptibility to urinary infection [16]. Thus, the observed relationship between diabetes duration and recurrent UTI may partly reflect the presence of chronic diabetes-related complications rather than an independent association with diabetes duration itself. However, the present study was not designed to determine mediation or establish a causal pathway.

Diabetic nephropathy remained independently associated with recurrent UTI after multivariable adjustment. Previous studies have reported associations between chronic kidney disease, diabetic nephropathy, and complicated or recurrent urinary infections [17]. Several mechanisms have been proposed to explain this relationship, including altered host defense, urinary tract dysfunction, greater healthcare exposure, and increased antimicrobial exposure. However, these mechanisms could not be directly evaluated in the present retrospective study. The observed association suggests that diabetic nephropathy may help identify patients who warrant closer assessment when recurrent urinary symptoms occur, although prospective studies are required to determine whether specific interventions in this population reduce recurrence.

Previous antibiotic exposure was independently associated with recurrent UTI, with patients exposed to antibiotics within the preceding 90 days demonstrating approximately twofold higher adjusted odds of recurrence. Previous antimicrobial exposure has been associated with subsequent isolation of resistant organisms and recurrent urinary infection in other clinical settings [18]. Alterations in the microbiota and selection of antimicrobial-resistant organisms have been proposed as possible explanations for this association. However, because antibiotic exposure may also reflect previous infections, healthcare utilization, or greater underlying disease burden, residual confounding cannot be excluded. Therefore, the present findings should be interpreted as an association between recent antibiotic exposure and recurrent UTI rather than evidence that antibiotic exposure directly causes recurrence. Nevertheless, the finding is consistent with the rationale for antimicrobial stewardship and supports appropriate culture-guided antimicrobial use whenever clinically feasible [19].

Urinary catheterization demonstrated the strongest independent association with recurrent UTI in the multivariable analysis. Previous studies have reported associations between urinary catheterization and healthcare-associated UTI, antimicrobial resistance, and recurrent urinary infection [20]. Catheter-associated biofilm formation, bacterial colonization, and disruption of normal urinary tract defenses are possible mechanisms underlying these observations. Because catheterization in this study may have reflected illness severity, hospitalization, or other underlying clinical conditions, confounding by indication cannot be excluded. Accordingly, the present findings do not establish catheterization as a direct cause of recurrent UTI. They do, however, support adherence to established catheter-management principles, including limiting catheter use to appropriate clinical indications and removing catheters when they are no longer required.

Nephrolithiasis was significantly associated with recurrent UTI in the univariable analysis but was not independently associated with recurrence after adjustment. Urinary calculi have been associated with recurrent urinary infection and may coexist with urinary obstruction, urinary stasis, mucosal injury, or persistent bacterial colonization [21]. The attenuation of the association after multivariable adjustment suggests that the observed unadjusted relationship may partly reflect other clinical factors. In particular, the relationship between nephrolithiasis, urinary obstruction, catheterization, and recurrent infection may be complex. Because the present study was not designed to evaluate these pathways, no causal inference can be made.

Regarding the microbial profile, *E. coli *was the predominant uropathogen in both recurrent and non-recurrent UTI groups, consistent with previous studies involving patients with diabetes mellitus. The distribution of individual organisms did not differ significantly between groups. In contrast, MDROs were significantly more frequent among patients with recurrent UTI and remained independently associated with recurrence after adjustment.

The association between MDRO isolation and recurrent UTI warrants cautious interpretation. The MDRO status was determined from the index UTI isolate and therefore preceded ascertainment of subsequent recurrence, strengthening the temporal interpretation of this finding. However, MDRO isolation may also be associated with previous antibiotic exposure, healthcare contact, urinary catheterization, and other characteristics that were not completely captured in the available records. Therefore, MDRO isolation should not be interpreted as a direct cause of recurrent UTI. Rather, it may represent a marker of a clinical profile characterized by greater antimicrobial exposure, healthcare utilization, or underlying complexity. The higher frequency of MDROs among patients with recurrent infection nevertheless has practical implications for antimicrobial selection and supports the value of urine culture and susceptibility testing when clinically indicated.

Dysuria and increased urinary frequency were the most common presenting symptoms in both groups, whereas urgency, suprapubic pain, fever, and flank pain were more frequent among patients with recurrent UTI. These differences provide a description of the clinical presentation of the index UTI but should not be interpreted as independent determinants of recurrence. The greater frequency of fever and flank pain among patients with recurrent UTI may indicate a higher proportion of complicated or upper urinary tract involvement, although the present study did not systematically classify infection according to anatomical site or clinical severity [22]. Therefore, this interpretation should be considered hypothesis-generating rather than causal.

The findings have several potential clinical implications. Patients with T2DM who have poor glycemic control, diabetic nephropathy, recent antibiotic exposure, urinary catheterization, or an MDRO isolated from the index UTI may represent a subgroup with a higher observed likelihood of recurrent infection. These characteristics may help clinicians identify patients who could benefit from closer follow-up and assessment of potentially modifiable clinical factors. Appropriate glycemic management, judicious antimicrobial prescribing, culture-directed treatment when indicated, avoidance of unnecessary urinary catheterization, and evaluation for underlying urinary tract abnormalities may be considered as components of comprehensive care. However, because this was an observational study, whether modification of these factors directly reduces recurrence cannot be determined from the present findings.

This study has several limitations. Its retrospective, single-center design limits the ability to establish temporal relationships and precludes definitive causal inference. The findings may therefore not be generalizable to other healthcare settings. Selection bias and residual confounding cannot be excluded despite multivariable adjustment. In particular, variables such as severity of acute illness, healthcare utilization, and indications for urinary catheterization may have influenced the observed associations.

Information regarding previous infections and antibiotic exposure depended on the completeness of medical records, and infections diagnosed or treated outside Fatima Memorial Hospital may not have been captured. Although previous antibiotic exposure was defined as systemic antibacterial therapy within 90 days before the index UTI, detailed information regarding antibiotic dose, adherence, and prior treatment response was not consistently available. Similarly, although MDRO status was determined from the index UTI isolate before ascertainment of subsequent recurrence, the retrospective design limits definitive assessment of the temporal and clinical relationship between antimicrobial resistance and recurrence. The possibility of overlap between factors contributing to recurrence and those identified during the index infection should therefore be considered when interpreting these associations.

Follow-up duration varied according to the timing of the index UTI during the study period. Patients with an index infection occurring near the end of the study period had less opportunity for a subsequent recurrence to be documented, which may have resulted in outcome misclassification. In addition, despite requiring documented clinical resolution or substantial improvement before counting a subsequent episode as a recurrence, the available records did not always permit reliable distinction between relapse and reinfection.

Furthermore, several potentially relevant variables, including menopausal status, sexual activity, post-void residual urine volume, sodium-glucose cotransporter-2 (SGLT2) inhibitor use, urinary incontinence, neurogenic bladder, adherence to diabetes treatment, detailed renal function measures, and structural urinary tract abnormalities, were not consistently available. In particular, the absence of SGLT2 inhibitor data represents a potentially important unmeasured pharmacological confounder because these agents promote glycosuria and may influence the risk of urinary and genital infections. Their absence, together with incomplete information on other clinical and pharmacological factors, may have contributed to residual confounding. These limitations should be considered when interpreting the observed associations, which should not be regarded as evidence of causality.

Despite these limitations, the study has several strengths, including the relatively large sample size, inclusion of culture-confirmed symptomatic UTIs, standardized assessment of microbiological findings, and direct comparison of recurrent and non-recurrent infections. Importantly, only the index UTI isolate was used to characterize baseline microbial and MDRO status, reducing the possibility that microbiological characteristics identified after recurrence were incorrectly treated as baseline covariates. The study therefore provides clinically relevant observational data on characteristics associated with recurrent UTI among patients with T2DM and may provide a basis for future prospective studies designed to establish temporal relationships and evaluate potential preventive strategies.

## Conclusions

Recurrent UTI was common among patients with T2DM and was independently associated with poor glycemic control, diabetic nephropathy, previous antibiotic exposure, urinary catheterization, and infection with multidrug-resistant organisms. *Escherichia coli* remained the predominant uropathogen in both recurrent and non-recurrent UTI groups, whereas MDROs were significantly more frequent among patients with recurrent infection. These findings may help clinicians identify patients at increased risk of recurrent UTI who may benefit from closer clinical monitoring, optimization of glycemic control, appropriate catheter management, and antimicrobial stewardship, including urine culture and susceptibility-guided antibiotic selection, particularly among patients with previous antibiotic exposure or suspected resistant infection. Given the observational design, these findings should be interpreted as associations rather than causal relationships. Further prospective multicenter studies are warranted to validate these associations and evaluate effective strategies for reducing recurrent UTIs among patients with diabetes.
